# Prognostic Value of Human Equilibrative NucleosideTransporter1 in Pancreatic Cancer Receiving Gemcitabin-Based Chemotherapy: A Meta-Analysis

**DOI:** 10.1371/journal.pone.0087103

**Published:** 2014-01-27

**Authors:** Zhu-Qing Liu, Ying-Chao Han, Xi Zhang, Li Chu, Jue-Min Fang, Hua-Xin Zhao, Yi-Jing Chen, Qing Xu

**Affiliations:** 1 Department of Medical Oncology, Shanghai Tenth People’s Hospital, Tongji University, School of Medicine, Shanghai, China; 2 Department of Spine Surgery, Shanghai East Hospital, Tongji University, School of Medicine, Shanghai, China; Sanjay Gandhi Medical Institute, India

## Abstract

**Background:**

The potential prognostic value of human equilibrative nucleoside transporter1 in pancreatic cancer receiving gemcitabine-based chemotherapy is variably reported.

**Objective:**

The objective of this study was to conduct a systematic review of literature evaluating human equilibrative nucleoside transporter1 expression as a prognostic factor in pancreatic cancer receiving gemcitabine-based chemotherapy and to conduct a subsequent meta-analysis to quantify the overall prognostic effect.

**Methods:**

Related studies were identified and evaluated for quality through multiple search strategies. Only studies analyzing pancreatic cancer receiving gemcitabine-based chemotherapy were eligible for inclusion. Data were collected from studies comparing overall, disease-free and progression-free survival (OS, DFS and PFS) in patients with low human equilibrative nucleoside transporter1 levels and those having high levels. The hazard ratio (HR) and its 95% confidence interval (95%CI) were used to assess the strength of associations. Hazard ratios greater than 1 reflect adverse survival associated with low human equilibrative nucleoside transporter1 levels.

**Results:**

A total of 12 studies (n = 875) were involved in this meta-analysis (12 for OS, 5 for DFS, 3 for PFS). For overall and disease-free survival, the pooled HRs of human equilibrative nucleoside transporter1 were significant at 2.93 (95% confidence interval [95% CI], 2.37–3.64) and 2.67 (95% CI, 1.87–3.81), respectively. For progression-free survival, the pooled HR in higher human equilibrative nucleoside transporter1 expression in pancreatic cancer receiving gemcitabine-based chemotherapy was 2.76 (95% CI, 1.76–4.34). No evidence of significant heterogeneity or publication bias was seen in any of these studies.

**Conclusion:**

These results support the case for a low human equilibrative nucleoside transporter1 level representing a significant and reproducible marker of adverse prognosis in pancreatic cancer receiving gemcitabine-based chemotherapy.

## Introduction

Pancreatic carcinoma, one of the most lethal malignancies, is the fourth leading cause of cancer-related deaths worldwide [1], partly due to resistance to most chemotherapeutic drugs. Inspite of recent surgical advances, the success rate remains unsatisfactory at 9% to 20% [2], [3]. Gemcitabine (GEM), the nucleoside pyrimidine analogue, is approved for use in non–small-cell lung cancer, breast cancer, and ovarian cancer. It is one of the most commonly used chemotherapeutic agents and is the single most effective agent in the palliation of advanced pancreatic cancer, where it has been shown to improve clinical symptoms and modestly extend survival [4]. However, treatment results and favorable outcomes with GEM remain variable. The response rate with GEM ranges from 5.4% to 16.7% [4], [5] in advanced or metastatic pancreatic cancer. GEM extended the median survival time (MST) of patients treated with 5FU from 4.2–4.5 months [4] to 5.9–6.5 months [5], [6] in locally advanced or metastatic pancreatic cancer. One large randomized phase III trial, the Charite Onkologie 001 (CONKO-001) study, demonstrated that in patients with complete resection of pancreatic cancer, the use of adjuvant gemcitabine for 6 months resulted in increased overall survival as well as disease-free survival [7]. The other large randomized phase III trial, the European Study Group for Pancreatic Cancer 3 (ESPAC-3) study, also confirmed the outcome [8]. Gemcitabine is strongly hydrophilic, and therefore, associated with slow passive diffusion through hydrophobic cellular membranes. Efficient permeation of gemcitabine across cell membranes requires specialized integral membrane transporter proteins [9]. Among these transporters, the human equilibrative nucleoside transporter 1(hENT1) is the major mediator of gemcitabine uptake into human cells [10]. Cells lacking hENT1 are highly resistant to gemcitabine [11].

Gemcitabine is a deoxycytidine analog, which crosses cell membrane through nucleoside transporters. Kinetic studies of human cell lines with defined nucleoside transporter processes have shown that gemcitabine intracellular uptake was mediated by hENT1, hENT2, hCNT1, and hCNT3, the hENT1 protein, which localizes in plasma and mitochondrial membranes, mediates the majority of gemcitabine transport in preclinical models [11]–[13]. The nucleoside transport inhibitors nitrobenzyl thioinosine or dipyridamole reduced sensitivity to gemcitabine by 39- to 1,800-fold [11]. Within the cell, gemcitabine is converted to its active diphosphate (dFdCDP) and triphosphate metabolites (dFdCDP). In this reaction, deoxycytidine kinase (dCK) is the rate-limiting enzyme, and cytidine deaminase (CDA) and 5′nucleotidase (5′-NT) are key rate-limiting enzymes [14]. The dFdCTP is incorporated into DNA with a subsequent addition of a natural nucleotide, thereby making the strand less vulnerable to DNA repair by base-pair excision [15]. However, the cytotoxicity is reinforced through several mechanisms. For example, dFdCDP inhibits ribonucleotide reductases (RRM1 and RRM2 subunits), which are the key enzymes in the synthesis of dNTP, inhibiting de novo DNA synthesis and repair pathways [16]. Decreased dCTP increases the rate of incorporation of dFdCTP into the DNA, to overcome the negative dCK feedback [17]. Chemoresistance of pancreatic cancer cell line to gemcitabine was related to the balance of dCK, RRM1, RRM2 and hENT1, which are the key enzymes involved in gemcitabine transportation and metabolic pathways [16].

Recently, low hENT1 was associated with poor prognosis in pancreatic cancer receiving gemcitabine-based chemotherapy (PCGC) [18]. Other studies showed no significant link between hENT1 and survival in PCGC [19]. However, both the studies involved a small sample size. We have, therefore, conducted a systematic review and meta-analysis to evaluate the overall risk of low hENT1 for survival in PCGC.

## Materials and Methods

### 1 Search strategy

A systematic literature search up to September2013 was performed in MEDLINE and EMBASE to identify relevant studies. An initial search strategy using recognized search terms [(hENT1 or human equilibrative nucleoside transporter1) and ‘prognosis’ and (‘pancreatic cancer’ or ‘pancreatic carcinoma’) and gemcitabine] was conducted.

### 2 Selection criteria

Studies were considered eligible if they met the following criteria: (i) measurement of pretreatment hENT1 values; (ii) evaluation of the potential association between pretreatment hENT1 and the survival outcome of PCGC; (iii) prospective or retrospective study design; and (iv) gemcitabine therapy. Articles were excluded based on the following criteria: (i) letters or review articles, (ii) laboratory studies, (iii) non-English or Chinese articles, or (iv) absence of key information such as sample size, hazard ratio (HR), 95% CI, and P value.

All searches were conducted independently by 2 reviewers (Z.L. and Y.H.).The studies identified were double-checked by both. Disagreements were resolved by consensus between the 2 reviewers or in consultation with a third reviewer (Q.X.). Additionally, a manual search was performed using references from the relevant literature, including all of the identified studies, reviews, and editorials. When duplicate studies were found, the study with reported HRs or involving additional patients (usually the most recent), was used for meta-analysis to prevent overlap between cohorts and overestimation of the overall HR.

### 3 Quality assessment

We systematically assessed the quality of all the studies included, according to a crucial review checklist of the Dutch Cochrane Centre proposed by MOOSE [20]. The key points of the current checklist include (i) clear definition of study population and origin of country; (ii) clear definition of study design; (iii) clear definition of outcome assessment, overall survival (OS), disease-free survival(DFS) and progression-free survival (PFS), with the failure event for DFS defined as disease relapse (local or regional), distant disease (including abdominal ascites, peritoneal seeding, and other abdominal sites), second primary or death from any cause; (iv) clear definition of cutoff for hENT1, and (v) sufficient period of follow-up. Studies disregarding all 5 of these points were excluded to ensure high quality of the meta-analysis.

**Figure 1:**
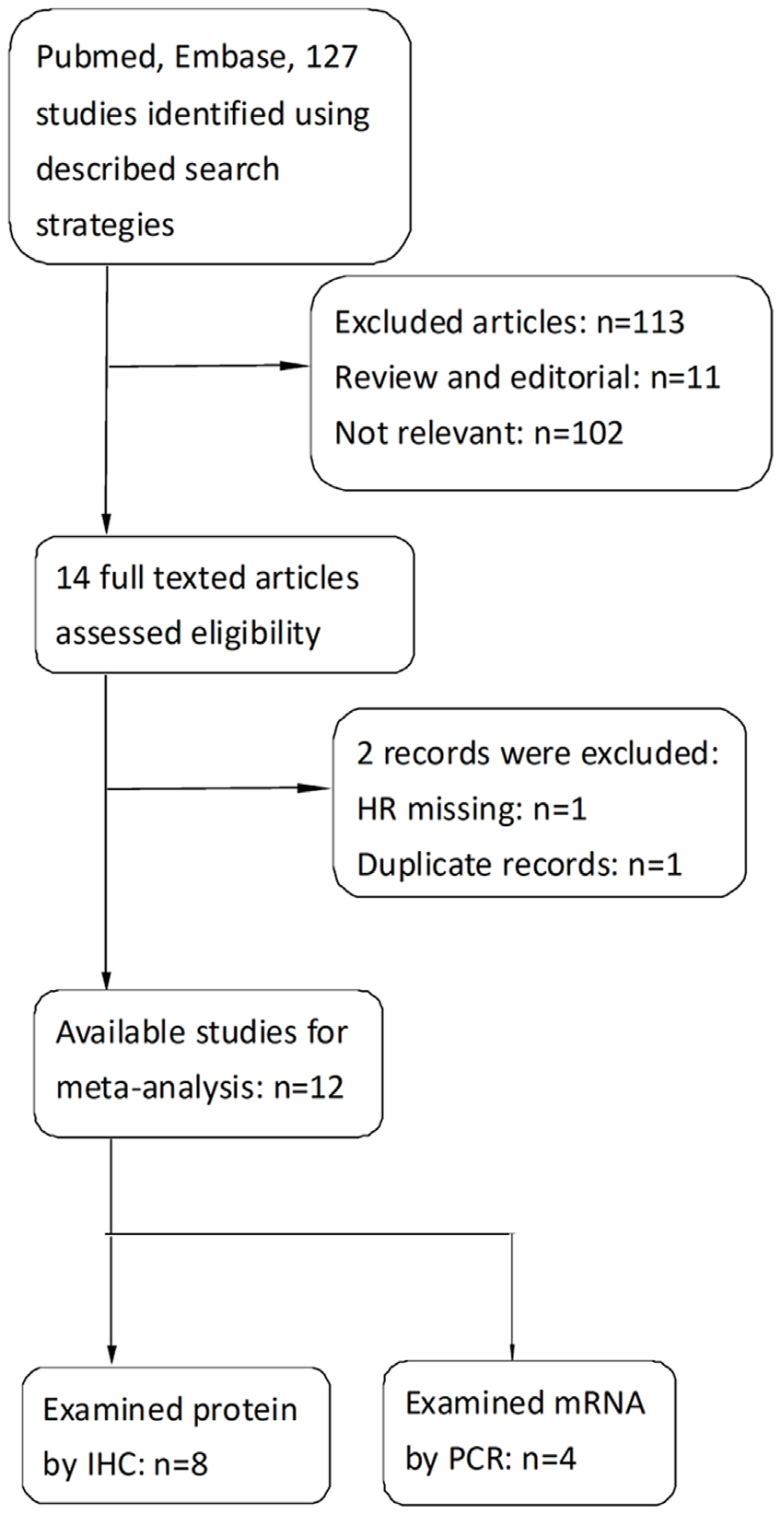
Flow diagram of the study selection process.

A flow diagram of the study selection process is showed in Figure 1.

### 4 Data extraction and conversion

The following data were collected: (i) publication details, including first author’s last name, year of publication, study population, country in which the study was performed; (ii) study design; (iii) characteristics of the studied population, including sample size, age, and the number of high expression; (iv) treatment setting, regime, measurement of the sample, and cutoff; and (v) HR of elevated hENT1 for OS, DFS and PFS as well as their 95% CIs. The simplest method consisted of the direct collection of HR and their 95% CIs from the original literature, with an HR of more than 1associated with a poor outcome. When these data were not directly reported, we extracted the total numbers of observed deaths and the numbers of patients in each group to calculate HR [21]. Data were extracted from the survival plots when data were only available as Kaplan-Meier curves, followed by estimation of the HR using the described method [21].

### 5 Statistical analysis

The heterogeneity of combined HRs was performed using Cochran’s Q test and Higgins’ I-squared statistic. A P value of less than 0.05 was considered significant. We used a random effects model (Der Simonian and Laird method) if heterogeneity was observed (P < 0.05). A fixed- effects model was applied in the absence of between-study heterogeneity (P ≥ 0.05). Publication bias was evaluated by the funnel plot with the Egger’s bias indicator test [22]. All analyses were conducted using the statistical software Stata (version 12.0).

## Results

### 1 Data retrieval

We identified 127 records for hENT1 after a primary search of PubMed and EMBASE. After reading titles and abstracts, 113 studies were excluded. Of the studies selected for detailed evaluation, 1 study was excluded as replicate [23] and 1 study was excluded due to missing HR data [24]. The final meta-analysis involved 12 studies for hENT1 [1], [18], [19], [25]–[33] (Fig. 1). Eight publications specifically involved two studies [18], [19], [26]–[30], [33].

### 2 Study characteristics

The characteristics of retained studies are summarized in Table 1. We collected data from 12 studies including a total of 875 patients with a median number of 55.5 patients per study (range  = 21–222). Five studies were conducted in Japan [19], [27]–[29], [32], 2 in the United States [18], [33], 1 in China [26], 1 in France [31] and 2 in Belgium [1], [30] and 1 in Italy [25]. Six articles stated the follow-up period, and clarified the median follow-up period. In the 12 studies (n = 875), values for hENT1 were analyzed by different means in each study. In 8 studies, hENT1 level was measured by immunohistochemistry (IHC). In the other 4 studies, hENT1 mRNA was measured by polymerase chain reaction (PCR). All of the articles related to IHC assessed and scored the hENT1 intensity. However, positive hENT1 staining in IHC was defined differently in various studies. Three of the IHC studies entailed a concordance analysis for hENT1 positivity with at least two observers, for 100% agreement. However no article reported the Kappa coefficients. In 9 studies,gemcitabine was used as adjuvant therapy, It was used as neoadjuvant therapy in one study and as palliative therapy in two other studies. Three of the studies were prospective analyses and 9 were retrospective analyses. Eleven of the selected studies presented HRs. In the remaining study, we calculated the HRs from the available data or survival curves.

**Table 1:** Summary of meta-analysis.

author	year	country	study design	recruitment period	age	case	treatment setting	gemcitabine-based regime	measurement	cutoff	High expression of hENT1	Survival analysis	HR(95% CI)	Follow-up months median (range)
Farrell JJ (18)	2009	US	P	1998–2002	-	91	adjuvant	Gemcitabine chemotherapy following chemoradiation after operation	IHC	No staining VS low and high staining	73	OS/PFS	report	-
Nakagawa N(27)	2013	Japan	RP	2002–2011	-	109	adjuvant	gemcitabine-based chemotherapy after operation	IHC	Low staining VS high staining	78	OS/DFS	report	39.7(2–122)
Murata Y (32)	2012	Japan	P	2005–2010	-	55	neoadjuvant	gemcitabine-based chemoradiotherapy before operation	IHC	Low staining VS high staining	39	OS	report	15(3.5–57.2)
Morinaga S (28)	2012	Japan	RP	2006–2008	64(45–74)	27	adjuvant	Gemcitabine	IHC	Low staining VS high staining	16	OS/DFS	report	-
Maréchal R (30)	2009	Belgium	P	2000–2003	56(34–83)	45	adjuvant	gemcitabine-based chemoradiation	IHC	Low staining VS high staining	19	OS/DFS	report	21.9(3.3–107.4)
Spratlin J (31)	2004	France	RP	1998–2002	58(39–72)	21	palliative	gemcitabine	IHC	Staining score 0 vs 1-2^+^	9	OS	report	-
Kim R (33)	2011	US	RP	2000–2005	66(45–93)	84	adjuvant	gemcitabine-based chemotherapy	PCR	0.2027	48	OS/PFS	report	60(44–110)
Eto K (19)	2013	Japan	RP	2007–2010	69(37–88)	56	palliative	gemcitabine-based chemotherapy	PCR	Median of the mRNA expression	33	OS/PFS	Survival curve	-
Fujita H (29)	2010	Japan	RP	1992–2007	-	40	adjuvant	gemcitabine-based chemotherapy	PCR	0.5	14	OS/DFS	report	-
Giovannetti E (25)	2006	Italy	RP	2001–2004	65(22–83)	81	Adjuvant/palliative	gemcitabine	PCR	1.23	37	OS	report	11.2(0.4–32.1)
Maréchal R (1)	2012	Belgium	RP	1996–2009	-	222	adjuvant	gemcitabine-based chemotherapy	IHC	Low staining VS high staining	86	OS	report	55.7(–)
Xiao JC (26)	2013	China	RP	2008–2009	61.4(38–80)	44	adjuvant	gemcitabine-based chemotherapy	IHC	No and low staining VS high staining	20	OS/DFS	report	-

P: prospective; RP: retrospective; OS: overall survival; PFS: progress free survival; DFS: disease-free survival;

IHC: immunohistochemistry; PCR: polymerase chain reaction ;( –)  =  not reported.

### 3 OS

**Figure 2:**
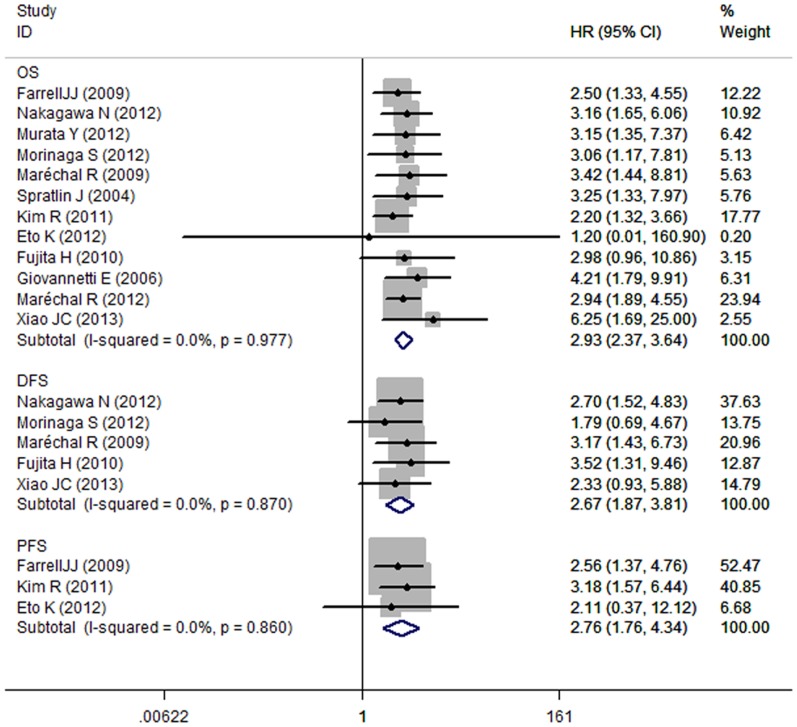
Forrest plots of studies evaluating hazard ratios (HR) with 95% confidence interval (95% CI) for low human equilibrative nucleoside transporter1 (hENT1) levels as compared with high levels. Survival data are reported as overall survival, disease-free survival, and progression-free survival.

Studies evaluating OS presented no evidence of significant heterogeneity for hENT1 (I^2^ = 0.0%, P = 0.977). Hence, a fixed- effects model was used to calculate a pooled HR and its 95% CI. The low hENT1 level was significantly correlated to OS with a pooled HR estimate of 2.93 (95% CI: 2.37–3.64) (Fig. 2).

In subgroup meta-analyses performed separately, the low hENT1 level was significantly correlated to OS with a pooled HR estimate of 3.06 (95% CI: 2.37–3.93) in IHC group. The pooled HR was 2.63 (95% CI: 1.75–3.97) in PCR group. The association of low hENT1 with OS in pancreatic cancer also did not differ by study location, study type, or treatment method (Table 2).

**Table 2:** Summary of risk estimates between low hENT1 and PCGC OS.

Stratification group	References	HR(95%CI)	Heterogeneity test	
			I^2^	P
All studies	1,18,19,25-32,33	2.93(2.37-3.64)	0.0%	0.977
Geographic region				
Asian	19,26-29,32	3.29(2.20-4.93)	0.0%	0.953
Europe	1,25,30,31,	3.21(2.30-4.48)	0.0%	0.906
US	18,33	2.32(1.57-3.43)	0.0%	0.754
Study type				
prospective	18,30,32	2.86(1.85-4.42)	0.0%	0.826
retrospective	1,19,25-29,31,33	2.96(2.31-3.79)	0.0%	0.910
Treatment method				
Adjuvant therapy	1,18,25-30, 33	2.90(2.31-3.65)	0.0%	0.896
Palliative therapy	19,31	3.14(1.30-7.58)	0.0%	0.692
hENT1 assay methodology				
IHC	1,18,26-28,30-32	3.06(2.37-3.93)	0.0%	0.978
PCR	19,25,29,33	2.63(1.75-3.97)	0.0%	0.621

IHC: immunohistochemistry; PCR: polymerase chain reaction; HR: hazard ratio; CI: confidence interval; hENT1: human equilibrative nucleoside transporter1

### 4 DFS and PFS

A fixed effects model was applied in the DFS and PFS analyses as the P values of between-study heterogeneity were 0.87 and 0.86. As illustrated in Figure 2, the combined HR of 2.67 (95%CI: 1.87–3.81) showed significant relationship between the low hENT1 level and the DFS in PCGC patients. The pooled HR was 2.76 (95% CI, 1.76–4.34) for low hENT1.

### 5 Publication bias

**Figure 3:**
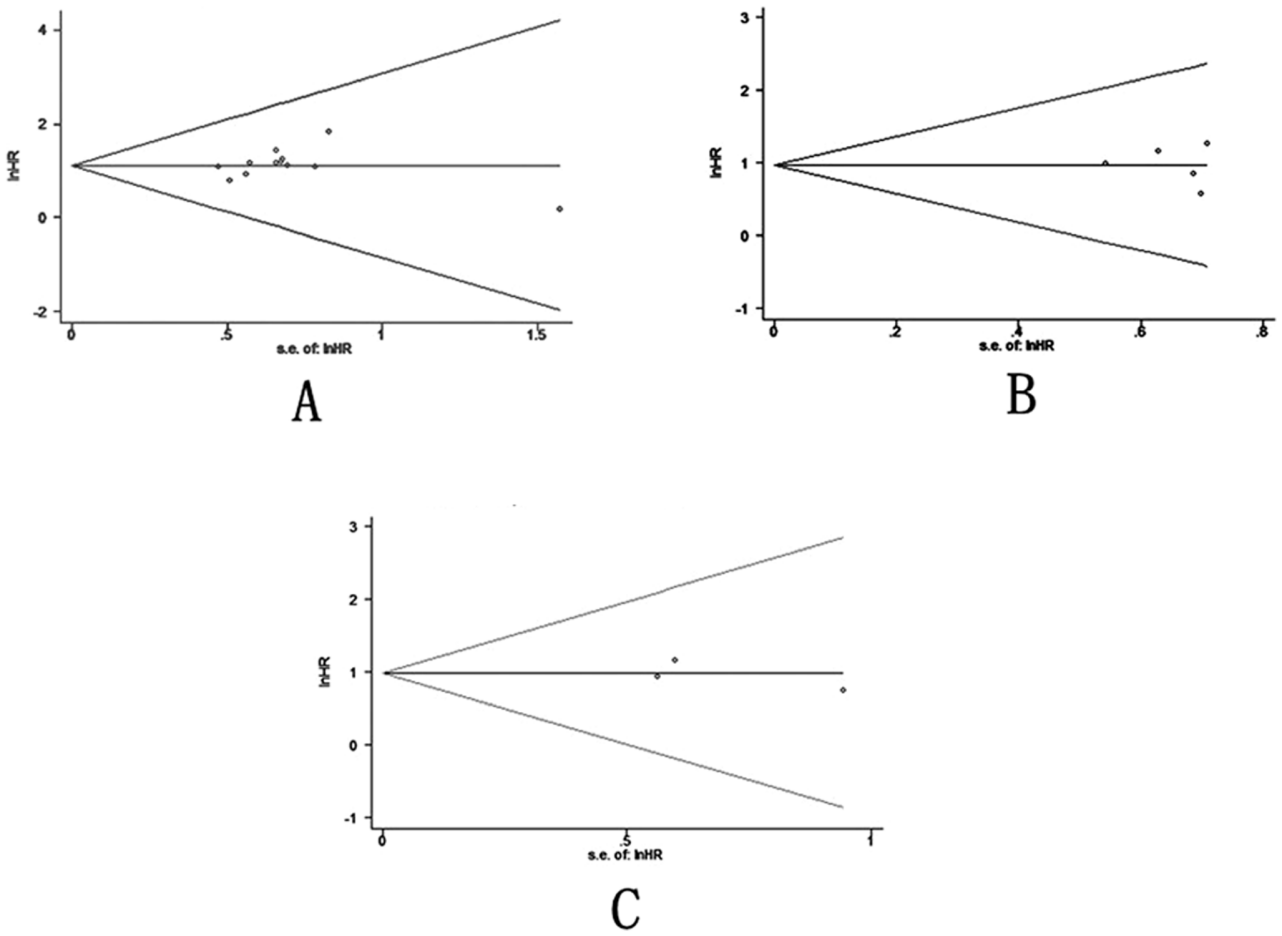
Funnel plots of studies included in the 3 meta-analyses. A) overall survival, B) disease-free survival, and C) progression-free survival.

Finally, we applied funnel plots and Egger’s test to evaluate publication bias of the included studies. As shown in Figure 3, all of the funnel plots were symmetrical. We observed no evidence of significant publication bias in OS, DFS and PFS, since the P values for Egger’s regression intercepts were more than 0.05 (P = 0.22, 0.769 and 0.707, respectively).

## Discussion

Previous meta-analyses of studies investigated the prognostic value of molecular markers in different malignancies. These include VEGF [34] and p53 [35]. To date, no such meta-analysis evaluated ENT1 in pancreatic cancer treated with gemcitabine. Furthermore, low hENT1 has been associated with poor prognosis in pancreatic cancer managed with gemcitabine-based chemotherapy [18]. Other studies have not shown any significant link between hENT1 and PCGC survival [19]. However, the number of patients included in each study was small. Therefore, it was essential to combine and analyze the data to obtain acceptable results.

In the present meta-analysis, we enrolled 12 studies related to the effects of low hENT1 expression on PCGC survival. In all these studies, hENT1 expression was detected by immunohistochemistry or PCR with surgical specimens. Meta-analysis suggested that low hENT1 was a factor associated with poor prognosis in PCGC. We further conducted subgroup analysis, in which hENT1 expression was measured by IHC. The results showed that low expression of hENT1 was closely associated with poor prognosis in patients with PCGC. Furthermore, hENT1 expression by PCR also showed significant impact on patients’ OS.

The recent PRODIGE 4/Accord 11 trial results have expanded the therapeutic options in metastatic PAC, by demonstrating the superiority of FOLFIRINOX regimen in comparison with gemcitabine alone [36]. This study included only patients who were aged below 76 years, with a good performance status (ECOG 0 or 1), no cardiac ischemia, and normal or nearly normal bilirubin levels. However, no study investigated whether this regimen or other regimens (fluoropyrimidines or erlotinib) were indicated for patients with low hENT1 expression in an adjuvant setting. Several studies reported methods involving histopathologic or cytopathologic diagnosis, including US- and CT-guided percutaneous biopsy, transpapillary pancreatic duct biopsy, and cytologic evaluation of pancreatic juice obtained via ERCP [37]-[39]. The ability to visualize small lesions with EUS is excellent, and, unlike other methods, the entire pancreas is readily imaged [37], [39], [40]. Thus, EUS-FNA is widely used as a cytological and histological sample collection tool in pancreatic cancer. Evaluation of hENT1 in pancreatic cancer tissue acquired with minimally invasive procedures (endoscopic ultrasound–guided fine-needle aspiration or computerized tomography–guided biopsy) warrants further study to determine the potential to individualize gemcitabine therapy in the majority of pancreatic cancer patients who present with locally advanced or metastatic disease.

Meta-analysis of prognostic literature is associated with a number of inherent limitations. Retrospective study design is one of the key limitations. Only three of the studies included in the current meta-analysis involve a prospective design. The availability and adequacy of corresponding clinicopathological data is also a significant consideration in retrospective studies of this type. We identified several studies reporting incomplete histopathological datasets. Other disadvantages include the following: First, we failed to review unpublished articles and abstracts, as most of the data were not required. Second, we included eligible English and Chinese studies only, suggesting a language bias. Third, HR calculation from data or extrapolation from survival curves in the articles, in the absence of directly reported HR values, introduced an element of decreased reliability.

Our meta-analysis also displayed significant strengths. First, the quality of studies included in the meta-analysis was satisfactory and strictly met the inclusion criteria. Second, the summary risk estimates of our study did not show any evidence of heterogeneity and publication bias. Third, we performed subgroup analysis by measuring hENT1.

## Conclusion

In conclusion, our meta-analysis indicated that low hENT1 expression was significantly associated with worse PCGC survival. hENT1 was a strong predictor of all the 3 survival outcomes. The critical role of hENT1 in cancer prognosis may contribute to its clinical utility. Considering the limitations of the present meta-analysis, further research with standardized, unbiased methods and larger, worldwide sample sizes are required to confirm our results.

## Supporting Information

Checklist S1 (DOC)
